# Intensive care unit length of stay can be longer due to the low serum vitamin D levels in patients with chronic obstructive pulmonary disease

**DOI:** 10.1186/2197-425X-3-S1-A184

**Published:** 2015-10-01

**Authors:** Ş Ö Keşkek, O Altınöz

**Affiliations:** Internal Medicine, Numune Training and Research Hospital, Adana, Turkey; Defne Hospital, Pulmonology, Antakya, Turkey

## Introduction

Vitamin D is an immune modulator hormone which has major effects on nearly all cells of the immune system. The deficiency of vitamin D has been found to be associated with many diseases. Chronic Obstructive Pulmonary Disease (COPD) is a chronic inflammatory pulmonary disease asociated with significant mortality and morbidity. Immune system disorders may aggravate the COPD due to the increased morbidity.

## Objectives

In this study we aimed to investigate the effect of serum vitamin d levels on COPD patients'duration of stay in intensive care unit (ICU).

## Methods

This retrospective study was performed in ICU of Adana Numune Training and Research Hospital. A total of 44 patients with COPD were included. Patients had diagnosed with COPD according to the GOLD 2011 report. The study and control groups included 23 and 21 patients with vitamin D deficiency or optimal vitamin D status, respectively. Serum 25(OH)D concentrations were used for vitamin D levels. Patients with different diseases in addition to the COPD were excluded. FEV1/FVC, FEV, serum creatinine levels and duration of stays in ICU were recorded and compared. MedCalc 15.2.2 (MedCalc Belgium) statistical software was used for statistical analysis.Chi-square is used to test the statistical significance of differences in frequencies. T test or Mann Whitney U tests were used for comparison of quantitative measurements between the two groups.

## Results

The groups were matched in terms of age and sex (p = 0.65, 0.931, respectively). FEV1/FVC, FEV1 and serum creatinine levels were comparable in both groups (p = 0.255, 0.393, 0.372, respectively). The levels of serum vitamin D were 14.7 ± 4.0 and 39.7 ± 11.1 in the study and control groups, respectively (p < 0.001). Duration of stay in ICU was longer in COPD patients with low serum vitamin D levels (9.1 ± 3.2 vs. 6.7 ± 4.1, p = 0.033).

## Conclusions

Vitamin D deficiency may aggravate COPD and lead to stay longer in ICU. Patients with COPD should be investigated for vitamin D deficiency. As an immune modulator vitamin D can protect COPD patients against co-morbidities such as infections. Therefore, supplementation of vitamin D can relieve the severity of disease and shorten the length of ICU stay.

## Grant Acknowledgment

The authors have no conflicts of interest.
